# Is Lone Hypertension a Risk Factor for More Severe COVID-19 Outcomes?

**DOI:** 10.5334/gh.1099

**Published:** 2022-03-01

**Authors:** Evgeniya V. Shalaeva, Alisher К. Shadmanov, Feruza L. Azizova, Khilola T. Mirakhmedova, Franz H. Messerli, Oscar H. Franco, Hugo Saner

**Affiliations:** 1 Tashkent Medical Academy, Tashkent, UZ; 2 Department for Cardiology, University Hospital Bern, Bern, CH; 3 Institute for Social and Preventive Medicine, University of Bern, CH

**Keywords:** COVID-19, SARS-CoV-2, Hypertension, JNC8 classification, mortality

## Abstract

**Background:**

Based on current evidence, it is not clear whether lone hypertension increases the risk for severe illness from COVID-19, or if increased risk is mainly associated with age, obesity and diabetes. The objective of the study was to evaluate whether lone hypertension is associated with increase mortality or a more severe course of COVID-19, and if treatment and control of hypertension mitigates this risk.

**Methods:**

This is a prospective multi-center observational cohort study with 30-day outcomes of 9,531 consecutive SARS-CoV-2 PCR-positive patients ≥ 18 years old (41.9 ± 9.7 years, 49.2% male), Uzbekistan, June 1-September 30, 2020. Patients were subclassified according to JNC8 criteria into six blood pressure stages. Univariable and multiple logistic regression was conducted to examine how variables predict outcomes.

**Results:**

The 30-days all-cause mortality was 1.18% (n = 112) in the whole cohort. After adjusting for age, sex, history of myocardial infarction (MI), type-2 diabetes, and obesity, none of six JNC8 groups showed any significant difference in all-cause mortality. However, age was associated with an increased risk of 30-days all-cause mortality (OR = 1.09, 95%CI [1.07–1.12], p < 0.001), obesity (OR = 7.18, 95% CI [4.18–12.44], p < 0.001), diabetes (OR 4.18, 95% CI [2.58–6.76], p < 0.001), and history of MI (OR = 2.68, 95% CI [1.67–4.31], p < 0.001). In the sensitivity test, being ≥ 65 years old increased mortality 10.56-fold (95% CI [5.89–18.92], p < 0.001). Hospital admission was 12.4% (n = 1,183), ICU admission 1.38% (n = 132). The odds of hospitalization increased having stage-2 untreated hypertension (OR = 4.51, 95%CI [3.21–6.32], p < 0.001), stage-1 untreated hypertension (OR = 1.97, 95%CI [1.52–2.56], p < 0.001), and elevated blood pressure (OR = 1.82, 95% CI [1.42–2.34], p < 0.001). Neither stage-1 nor stage-2 treated hypertension patients were at statistically significant increased risk for hospitalization after adjusting for confounders. Presenting with stage-2 untreated hypertension increased the odds of ICU admission (OR = 3.05, 95 %CI [1.57–5.93], p = 0.001).

**Conclusions:**

Lone hypertension did not increase COVID-19 mortality or in treated patients risk of hospitalization.

## Introduction

Hypertension has been identified as the most common comorbidity among COVID-19 patients [1]. Hypertension becomes more prevalent with age and is often associated with other risk factors such as obesity, diabetes, hyperlipidemia [1,2]. Evidence suggests also that older patients and patients with comorbidities such as obesity, diabetes or cardiovascular diseases may have a more severe course of COVID-19 [3]. Severe COVID-19 is defined as hospitalization, admission to the ICU, intubation or mechanical ventilation, or death [3]. A history of hypertension has been shown to increase odds of hospitalization and severe illness twofold. A similar increase was seen with diabetes and chronic lung disease (OR = 2.2), fever (OR = 2.89) and, obesity (OR = 1.8–3.7) [2,3,4,5,6]. The presence of hypertension has also been associated with longer hospital stays, and intubation [5,7]. However, it is still under investigation whether arterial hypertension per se is a risk factor for a more severe course of COVID-19 disease and whether control of hypertension mitigates this risk compared to uncontrolled hypertension.

The aim of this study was to evaluate whether lone hypertension is associated with increased mortality or a more severe course of COVID-19, and if treatment and control of hypertension mitigates this risk. We hypothesized that lone hypertension is associated with increased mortality or a more severe course of COVID-19, and that treatment and control of hypertension mitigates this risk compared to uncontrolled hypertension.

## Methods

### Study design

This is a prospective multi-center observational cohort study with 30-day outcomes from 12,948 consecutive patients with confirmed a SARS-CoV-2 PCR-positive. Patients had first medical evaluation in the Tashkent COVID-19 distribution centers, with 30 day follow-up after treatment in 46 outpatient state clinics of Tashkent, the capital of Uzbekistan, during the period of June 1-September 30, 2020.

### Study patients

In this study, 12,948 consecutive patients with baseline characteristics of all confirmed COVID-19 patients were included and data were retrieved on the day of the first visit to the Tashkent COVID-19 distribution centers after the onset of clinical symptoms. Of them, 9,531 patients were ≥ 18 years old (41.9 ± 9.7years, 49.2% male), and were included in the data analysis. Patients who were seeking medical care through emergency services and people using the private health care sector were not included in the study due to separate regulations for data availability and surveillance systems.

### Study setting

Before June 1, 2020, all patients with the confirmed COVID-19 status based on RT-PCR were immediately admitted to the hospitals regardless of symptoms. Since June 1, the management of COVID-19 patients was organised at the COVID-19 distribution centers due to progressively increased number of infected patients and overload of hospitals beds allocated to treat COVID-19 patients. This was done in accordance with the Presidential Decree No. R-5537, which was aimed to combat COVID-19 in Uzbekistan [8]. After exam, CT scan, and laboratory tests, if mild COVID-19 course, patients were referred to outpatient clinics; if moderately severe, patients might stay under the supervision of medical personnel for 1–5 days; if severe, patients were admitted to the COVID-19 hospitals. ICU units had been organized at the distribution centers for patients who required critical care.

The active surveillance and follow-up response system was established on the basis of outpatient clinics to monitor patients’ conditions daily until symptoms resolved for outpatients and within at least 30 days after dispatch from distribution centers or hospitals. Management of COVID-19 patients in hospital, at the distribution centers, and outpatient clinics, including testing, exams, and medications was completely free of charge for patients and covered by the government of Uzbekistan.

### Diagnosis and clinical classification of COVID-19 infection

All patients with COVID-19 infection diagnosis met the following criteria: positive SARS-COV-2 RNA by RT-PCR, plus fever, respiratory and/or other symptoms. In case of abnormal lung sounds, chest CT scan or X-ray was performed. The clinical classification of patients was based on the Interim Guidelines for the Management of Patients Infected with COVID-19 (7th ed.) developed by the Ministry of Health of Uzbekistan, adopted from the temporary guidelines of the WHO [9]. Clinical classification of the COVID-19 course was the following: 1) mild clinical symptoms; 2) moderately severe patients who had not signs of severe COVID-19; 3) severe course with signs of respiratory distress, respiratory rate ≥ 30 beats/min, mean oxygen saturation ≤ 93% at rest; 4) critically severe patients were defined if intensive or critical care treatment, e.g. mechanical ventilation, vasopressor therapy required.

### Data acquisition

Baseline characteristics of confirmed COVID-19 patients were retrieved on the day of the exam at the COVID-19 distribution centers. Patient’s risk factors such as obesity, history of cardiovascular disease (CVD), history of diabetes, chronic obstructive pulmonary disease (COPD) or asthma were assessed along with socio-economic factors, lifestyle risk factors such as self-reported smoking status, physical activity, and nutrition. Diabetes was defined by a hemoglobin HbA1C ≥ 6.5%, history of physician based diagnosis, or use of anti-diabetic medications according to 2019 ESC Guidelines on diabetes, pre-diabetes, and cardiovascular diseases [10]. Smoking was defined as current (tobacco products used within the last month), occasional or never [11]. Coronary artery disease was defined according to the 2019 ESC Guidelines for the diagnosis and management of chronic coronary syndromes [11]. In adults (age over 18 years) obesity was defined by a BMI ≥ 30 kg/m [2,12]. Lungs CT scan was conducted for suspected pneumonia.

### Hypertension criteria/definition

Blood pressure (BP) was measured after some rest, in sitting position, at the beginning and at the end of the healthcare providers exam in both arms, the mean of two measurements was used [3,13,14]. The main analysis was conducted based on BP and history of antihypertensive therapy. Patients were subdivided into six hypertension groups according to guidelines of the Eighth Joint National Committee (JNC8) [13]:

- (1) Normal: Less than 120/80 mm Hg;
- (2) Elevated: Systolic between 120–129 *and* diastolic less than 80;
- Stage 1: Systolic between 130–139 *or* diastolic between 80–89; (3) *treated; (4) untreated*
- Stage 2: Systolic at least 140 *or* diastolic at least 90 mm Hg; (5) *treated; (6) untreated*

Patients were considered treated if they had previously established diagnosis of arterial hypertension, and had received prescriptions of medications within 90 days before visiting the outpatient clinic due to COVID-19 symptoms. The patients’ hypertension treatment of antihypertensive medications, such as angiotensin converting enzyme inhibitors (ACEI), angiotensin receptors blockers (ARB), calcium channel blockers (CCB), beta-blockers (BB), diuretics and others were recorded. The patient was considered untreated if hypertension was not previously diagnosed (based on the patient history/charts), if the patient was aware of having hypertension but did not take prescribed medications or used ad hoc only in case of ‘emergency’. (***Table 1***).

**Table 1:** Cohort of COVID-19 patients with the 30-day outcome subclassified according to JNC8 criteria into six blood pressure stages (n = 9,531).


	TITLE OF THE GROUP JNC8	BLOOD PRESSURE SYSTOLIC/DIASTOLIC, MMHG	N	%

1	Normal	<120/<80	6697	70.3

2	Elevated	120–129/<80	568	6.0

3	Stage-1 untreated^	130–139/80–89	457	4.8

4	Stage-1 treated*	130–139/80–89	711	7.5

5	Stage-2 untreated^	>40/>90	233	2.4

6	Stage-2 treated*	>140/>90	865	9.1

		Total	9,531	100


* Patients were considered as treated if had previously established diagnosis of arterial hypertension, had antihypertensive prescription, and consumed medications within 90 days before the outpatient visit. ^ Patient were considered untreated if hypertension was not previously diagnosed (based on the patient history/charts), if the patient was aware of having hypertension but did not take prescribed medications or used ad hoc only in case of ‘emergency’.

### Outcomes

We specified three outcomes that represented severe cases of COVID-19: 1) Hospital admission for one or more nights. 2) ICU admission for one or more nights. 3) All-cause mortality within 30 days. Patients’ survival/death was confirmed by the follow-up data of the outpatient clinics as well as with vital statistics.

### Statistical analysis

Statistical analyses were performed using SPSS software (v27, IBM, Chicago, IL, USA). Descriptive statistics for studied variables are presented as mean±SD (standard deviation) for normally distributed continuous variables, median with interquartile range for non-normally distributed continuous variables and frequency with percentage for categorical variables. Variables were compared with independent Student *t*-tests for normally distributed continuous data, and Chi-square test for categorical data. Differences between groups were determined by a one-way analysis of variance (ANOVA), with a subsequent Tukey’s/Dunnet C post hoc test. There were no loss of follow-up unless patients death.

The association of risk factors and hospital admission as well as 30-day all-cause mortality was assessed using multivariate logistic regression to examine how variables predict an outcome in the main analysis (six hypertension groups based on the JNC8) [11]. Univariable analysis of each risk factor (group of arterial hypertension, age, sex, history of obesity, diabetes, obstructive CVD, prior cases of acute coronary syndrome, e.g. myocardial infarction (MI), history COPD or asthma) was assessed separately. In the multivariable analysis, we adjusted all selected covariates with the reference for the categorical variable as normal blood pressure, no obesity, no diabetes, no MI, etc). Variables used in the analysis did not have missing data. To bring statistical power we analyzed 3,087 patients who were 50 years old and older. The statistical test did not show any significant difference for any stage of hypertension, thus the results are presented for the entire cohort.

### Sensitivity analyses

We also conducted a sensitivity analysis to show the differences on the outcomes between the presence vs. no hypertension per se (Supplement 1–3). We also tested the hypotheses whether controlled or uncontrolled hypertension compare with no hypertension were associated with severe COVID-19. Controlled means BP < 140/90 during study visit or hospitalization and irrespective of treatment. Treatment was used only within the definition of hypertension (Supplement 4–7). We also conducted additional regression analysis stratified by gender (Supplement 8). Univariable and multivariable logistic regression to examine the association between 30-day mortality and baseline characteristics in COVID-19 patients is shown in the Supplement 9.

## Results

The study population consisted of 9,531 consecutive patients with confirmed COVID-19 infection. Over 96% of patients had a first medical contact within two days of onset of symptoms. ***Table 1*** represents the distribution of the patients with COVID-19 divided into six hypertension groups according to JNC8 as per methods [11]. Of the entire cohort, 2,442 out of 9,531 (25.6%) patients had hypertension, of them 1,344 (14.1%) were controlled (BP < 140/90 mmHg), and 1,098 (11.5%) uncontrolled (BP ≥ 140/90 mmHg). The prevalence of stage-1 and stage-2 hypertension was 2,266 out of 9,531 patients (23.8%). ACEI inhibitors were most commonly prescribed antihypertensive medications, followed by ARBs and BBs (***Table 2***).

**Table 2:** Baseline characteristics of COVID-19 patients with the 30-day outcome (n = 9,531). Hypertension was subclassified according to JNC8 criteria into six blood pressure stages (n = 9,531).


	NORMAL N = 6,697	ELEVATED N = 568	STAGE-1 UNTREATED N = 457	STAGE-1 TREATED N = 711	STAGE-2 UNTREATED N = 233	STAGE-2 TREATED N = 865	TOTAL N = 9,531

**Age (years), mean** ± **SD**	35.9 ± 13.1	43.4 ± 11.3	51.8 ± 10.9	57.3 ± 10.1	61.4 ± 7.7	65.9 ± 9.7	41.9 ± 9.7

**Age ≥ 65 years, n (%)**	236 (3.5)	23 (4.0)	44 (9.6)	143 (20.1)	85 (35.6)	428 (49.5)	959 (10.1)

**Sex, n (%)**							

Male	3347 (50.0)	305 (53.7)	209 (45.7)	300 (42.2)	109 (46.8)	420 (48.6)	4,689 (49.2)

Female	3350 (50.0)	263 (46.3)	248 (54.3)	411 (57.8)	124 (53.2)	445 (51.4)	4,842 (50.8)

**Course of COVID-19 disease**							

Mild	3,677 (54.9)	232 (40.8)	159 (34.8)	298 (41.9)	11 (4.7)	343 (39.7)	4,720 (49.5)

Moderately severe	2,599 (38.8)	212(37.3)	135 (29.5)	281 (39.5)	72 (30.9)	331 (38.3)	3,630 (38.1)

Severe	401 (5.9)	116 (20.4)	153 (33.4)	110 (15.4)	122 (52.3)	147 (17.0)	1,049 (11.0)

Critical (ICU admission)	20 (0.3)	8 (1.41)	10 (2.2)	22 (3.1)	28 (12.02)	44 (5.1)	132 (1.38)

**Comorbidities**							

**History of myocardial infarction**	136 (2.0)	36 (6.3)	44 (9.6)	73 (10.3)	39 (16.7)	206 (24.0)	534 (5.6)

**Obesity**	575 (8.6)	225 (39.6)	156 (34.1)	255 (35.2)	125 (53.6)	350 (40.5)	1,686 (17.7)

**Diabetes**	262 (3.9)	65 (11.4)	115 (25.2)	155 (21.8)	115 (49.5)	310 (35.8)	1,022 (10.7)

**Antihypertensive medications**							

ACEI	189 (2.8)	141 (24.8)	92 (20.1)	435 (61.2)	42 (18.0)	503 (58.2)	1,387 (14.6)

ARB	96 (1.4)	45 (7.9)	71 (15.5)	198 (27.8)	35 (15.0)	395 (45.7)	840 (8.8)

CCB	49 (0.7)	38 (6.7)	30 (6.6)	87 (12.2)	18 (7.7)	152 (17.6)	374 (3.9)

BB	339 (5.1)	86(15.1)	66 (14.4)	275 (38.7)	39 (16.7)	407 (47.1)	1,192 (12.5)

Diuretics	121 (1.8)	25 (4.4)	32 (7.0)	48 (6.8)	16 (6.9)	88 (10.2)	330 (3.5)

**Outcomes:**							

**Duration of COVID-19, days** ^1^	8.7 ± 6.0	12.9 ± 7.8	14.9 ± 9.3	12.4 ± 7.7	21.9 ± 9.4	14.6± 8.2	10.4 ± 7.3

**Hospitalisation**	424 (6.33)	120 (21.13)	129 (28.23)	142 (19.9)	144 (61.8)	224 (25.9)	1,183 (12.4)

Stay in hospital, days	9.4 ± 3.7	9.4 ± 3.8	9.3 ± 2.6	12.3 ± 4.7	11.4 ± 3.7	12.1 ± 5.0	10.5 ± 4.2

Stay in ICU, days	3.6 ± 3.4	3.4 ± 1.2	2.8 ± 1.3	4.1 ± 2.2	5.3 ± 3.1	5.0 ± 3.6	4.4 ± 3.1

**All-cause mortality 30-days**	13 (0.19)	8 (1.41)	0	18 (2.53)	14 (6.01)	59 (6.82)	112 (1.18)


Data are n (%), where n is the number of participants with non-missing data, or mean ± SD. Baseline characteristics were calculated for the participants at the first outpatient exam. ^1^ COVID-19 symptoms onset to recovery or symptoms onset to death. ACEI = Angiotensin-converting enzyme inhibitors. ARB = Angiotensin-receptor blockers. BB = Beta-blockers. CCB = Calcium channel blockers CI = confidence interval. ICU = intensive care unit.

Age gradually increased from patients with normal BP to stage-2 hypertension mostly due to higher prevalence of people 65 years old and older (p < 0.001). A similar tendency between groups was observed for patients with obstructive CAD (p < 0.001). Patients from the hypertension stage-2 untreated group had greater prevalence of diabetes, obesity and/or symptomatic CAD (***Table 2***).

### COVID-19 Hospital Admission

During the course of COVID-19, 1,183 out of9,531 patients (12.41%) required at least one day of in-hospital treatment. Patients with stage-2 untreated hypertension had the highest hospitalization rate among all groups with 144 out of 233 (61.8%), following by stage-1 untreated with 129 out of 457 (28.2%), and stage-2 treated group with 224 out of 865 (25.9%). The hospital stay was longer in patients with stage-2 hypertension, and stage-1 treated (one-way ANOVA, was significant, F (5, 1177) = 24.3, p < 0.001, for pairwise combinations).

The multivariable logistic regression analysis was conducted to present unadjusted and the adjusted effect of confounders (six hypertension groups according to JNC8 [11], age, sex, history of myocardial infarction, type-2 diabetes, obesity) to the hospital.

In the unadjusted model, all hypertension groups had significantly increased odds to be hospitalized with COVID-19 compared to the patients with normotensives (reference group). The greatest odds were in the case of having stage-2 untreated hypertension (OR = 23.91, 95%CI [18.03–31.7]), following by stage-2 treated and stage-1 untreated.

After adjusting for covariates, having stage-2 untreated hypertension remained significant and increased the odds of hospitalization 4.51-fold vs. normotensive patients (OR = 4.51, 95% CI [3.21–6.32], p < 0.001), stage-1 untreated hypertension (OR = 1.97, 95%CI [1.52–2.56]), and elevated BP group (OR = 1.82, 95%CI [1.42–2.34]) (***Table 3***). Stage 1 and 2 treated hypertension did not show statistically significant effect on hospitalization after adjusting for the confounders (***Table 3***). The greater odds of hospital admission in patients with untreated hypertension may be explained by greater prevalence of obesity, symptomatic CAD, and diabetes, as well as the fact that the patient who were aware of having hypertension but did not take prescribed medications or used ad hoc only in case of ‘emergency’ were included as untreated patients.

**Table 3:** Association between hospital admission and hypertension subclassified into six blood pressure stages according to JNC8 criteria in COVID-19 patients adjusted for confounders, multivariable logistic regression (n = 9,531).


	AVAILABLE DATA IN THE UNIVARIABLE ANALYSIS N = 9,531	PATIENTS WHO WERE HOSPITALIZED N = 1,183	UNIVARIABLE		MULTIVARIABLE	

			CRUDE ODDS RATIO (95% CI)	P VALUE	ADJUSTED ODDS RATIO (95% CI)	P VALUE

**Arterial Hypertension**						

Normal	6,697	424 (6.3)	1 (ref)		1 (ref)	..

Elevated	568	120 (21.1)	3.95 (3.16–4.95)	< 0.001	1.82 (1.42–2.34)	<0.001

Stage-1 untreated	457	129 (28.2)	5.81 (4.63–7.29)	< 0.001	1.97 (1.52–2.56)	<0.001

Stage-1 treated	711	142 (19.9)	3.68 (2.99–4.54)	< 0.001	0.91 (0.71–1.18)	0.492

Stage-2 untreated	233	144 (61.8)	23.91 (18.03–31.7)	< 0.001	4.51 (3.21–6.32)	<0.001

Stage-2 treated	865	224 (25.9)	5.17 (4.31–6.19)	< 0.001	0.78 (0.61–1.01)	0.061

**Age, years**	9,531		1.05 (1.05–1.06)	< 0.001	1.03(1.026–1.04)	<0.001

**Sex**						

Male	4,689	545 (11.6)	1 (ref)		1 (ref)	..

Female	4,842	638 (13.2)	1.15 (1.02–1.30)	0.018	0.93 (0.98–1.30)	0.107

**Myocardial infarction**						

No	8,612	985 (11.4)	1 (ref)		1 (ref)	..

Yes	919	198 (21.5)	4.79 (3.98–5.78)	< 0.001	1.28 (0.98–1.30)	0.058

**Obesity**						

No	7,845	503 (6.4)	1 (ref)		1 (ref)	..

Yes	1,686	680 (40.3)	9.86 (8.63–11.26)	< 0.001	5.54 (4.78–6.44)	<0.001

**Diabetes**						

No	8,509	735 (8.6)	1 (ref)		1 (ref)	..

Yes	1,022	448 (43.8)	8.26 (7.14–9.54)	< 0.001	3.35 (2.77–4.05)	<0.001


Data are n (%) unless otherwise specified. CI = confidence interval.

The adjusted effect of age to increase chances of COVID-19 hospitalization remained statistically significant (OR = 1.03, 95%CI [1.03–1.04]). Obesity (having BMI > 30) increased odds for being hospitalized 5.54-fold (OR = 5.54, 95%CI [4.78–6.45]), type-2 diabetes 3.35-fold (OR = 3.35, 95% CI [2.77–4.05]), and history of myocardial infarction 1.28-fold (OR = 1.28, 95%CI [0.98–1.3]).

### COVID-19 ICU Admission

In the whole cohort 132 patients (1.38%) required at least 1 day of stay in the ICU. After adjusting for age, sex, history of myocardial infarction, type 2 diabetes, obesity, COPD/asthma, only having stage 2 untreated hypertension increased the odds of hospitalization (OR = 3.05, 95%CI [1.57–5.93]). Neither stage 1 or 2 treated hypertension patients or stage 1 untreated were at statistically significant increased risk for ICU admission after adjusting for confounders (***Table 4***). The adjusted effect of covariates on ICU admission remained significant: age (OR = 1.07, 95%CI [1.05–1.09]), obesity (OR = 5.61, 95%CI [3.55–8.88]), diabetes (OR = 4.19, 95% CI [2.74–6.40]), and history of myocardial infarction (OR1.76-fold (95%CI [1.11–2.79]).

**Table 4:** Association between ICU admission and hypertension subclassified into six blood pressure stages according to JNC8 criteria in COVID-19 patients adjusted for confounders, multivariable logistic regression (n = 9,531).


	AVAILABLE DATA IN THE UNIVARIABLEANALYSIS N = 9,531	PATIENTS ADMITTED IN THE ICU N = 132	UNIVARIABLE		MULTIVARIABLE	

			CRUDE ODDS RATIO (95% CI)	P VALUE	ADJUSTED ODDS RATIO (95% CI)	P VALUE

**Arterial Hypertension**						

Normal	6,697	20 (0.3)	1 (ref)		1 (ref)	..

Elevated	568	8 (1.4)	4.77 (2.09–10.88)	<0.001	1.58 (0.65 = 3.86)	0.309

Stage-1 untreated	457	10 (2.2)	7.47 (3.48–16.05)	<0.001	1.75 (0.79 = 3.85)	0.165

Stage-1 treated	711	22 (3.1)	10.66 (5.79–19.63)	<0.001	1.23 (0.62 = 2.43)	0.555

Stage-2 untreated	233	28 (12.0)	45.59 (25.26–82.29)	<0.001	3.05 (1.57 = 5.93)	0.001

Stage-2 treated	865	44 (5.1)	17.89 (10.49–30.50)	<0.001	0.95 (0.51 = 1.79)	0.880

**Age, years**	9,531		1.10 (1.09–1.12)	<0.001	1.07 (1.05 = 1.09)	< 0.001

**Sex**						

Male	4,689	63 (1.3)	1 (ref)		1 (ref)	..

Female	4,842	69 (1.4)	1.06 (0.75–1.49)	0.734	0.86 (0.59 = 1.26)	0.441

**Myocardial infarction**						

No	8,612	88 (1.0)	1 (ref)		1 (ref)	..

Yes	919	44 (4.8)	9.09 (6.26–13.20)	<0.001	1.76 (1.11 = 2.79)	0.016

**Obesity**						

No	7,845	27 (0.3)	1 (ref)		1 (ref)	..

Yes	1,686	105 (6.2)	19.23 (12.56–29.45)	<0.001	5.61 (3.55 = 8.88)	< 0.001

**Diabetes**						

No	8,509	43 (0.5)	1 (ref)		1 (ref)	..

Yes	1,022	89 (8.7)	18.78 (12.97–27.19)	<0.001	4.19 (2.74–6.40)	< 0.001


Data are n (%) unless otherwise specified. CI = confidence interval. ICU = intensive care unit.

### Thirty-day All-cause Mortality

In the whole cohort, all-cause mortality was 1.18% (112 out of 9,531). In the JNC8 stages mortality was 0.19% (n = 13) in normotensives, 1.41% (n = 8) in stage elevated, zero in stage-1 untreated, 2.53% (n = 18) in stage-1 treated, 6.01% (n = 14) in stage-2 untreated and 6.82% (n = 59) in stage-2 treated (Chi-square = 356.75; p < 0,001). After adjusting for age, sex, history of MI, type-2 diabetes, obesity, none of six JNC8 groups showed any significant difference in 30-day all-cause mortality (***Table 4***). However, age was associated with an increased risk of 30-day all-cause mortality (OR = 1.09, 95%CI [1.07–1.12], p < 0.001), obesity (OR = 7.18, 95%CI [4.18–12.44], p < 0.001), diabetes (OR 4.18, 95%CI [2.58–6.76], p < 0.001), and history of MI (OR = 2.68, 95%CI [1.67–4.31], p < 0.001). In the sensitivity test, being ≥ 65 years old increased mortality 10.56-fold (OR = 10.56, 95%CI [5.89–18.92], p < 0.001) (***Table 5***).

**Table 5:** Association between 30-day mortality and hypertension subclassified into six blood pressure stages according to JNC8 criteria in COVID-19 patients adjusted for confounders, multivariable logistic regression (n = 9,531).


	AVAILABLE DATA IN THE UNIVARIABLE ANALYSIS N = 9,531	PATIENTS WHO DIED N = 112	UNIVARIABLE		MULTIVARIABLE	
	
			CRUDE ODDS RATIO (95% CI)	P VALUE	ADJUSTED ODDS RATIO (95% CI)	P VALUE

**Arterial Hypertension**						

Normal	6,697	13 (0.2)	1 (ref)		1 (ref)	..

Elevated	568	8 (1.4)	7.35 (3.03–17.79)	<0.001	2.49 (0.94–6.64)	0.068

Stage-1 untreated	457	0	0	0.994	0	0.992

Stage-1 treated	711	18 (2.5)	13.35 (6.52–27.37)	<0.001	1.25 (0.56–2.81)	0.590

Stage-2 untreated	233	14 (6.0)	32.87 (15.27–70.76)	<0.001	1.73 (0.74–4.02)	0.203

Stage-2 treated	865	59 (6.8)	37.63 (20.55–68.92)	<0.001	1.49 (0.75–2.99)	0.258

**Age, years**	9,531		1.13 (1.11–1.15)	<0.001	1.09 (1.07–1.12)	< 0.001

**Sex**						

Male	4,689	48 (1.0)	1 (ref)		1 (ref)	..

Female	4,842	64 (1.3)	1.29 (0.89–1.89)	0.178	0.97 (0.63–1.51)	0.909

**Myocardial infarction**						

No	8,612	57 (0.7)	1 (ref)		1 (ref)	..

Yes	919	55 (5.9)	18.01 (12.29–26.38)	<0.001	2.68 (1.67–4.31)	< 0.001

**Obesity**						

No	7,845	18 (0.2)	1 (ref)		1 (ref)	..

Yes	1,686	94 (5.6)	25.68 (15.46–42.63)	<0.001	7.18 (4.18–12.44)	< 0.001

**Diabetes**						

No	8,509	31 (0.4)	1 (ref)		1 (ref)	..

Yes	1,022	81 (7.9)	23.54 (15.48–35.81)	<0.001	4.18 (2.58–6.76)	< 0.001


Data are n (%) unless otherwise specified. CI = confidence interval.

Univariable and multivariable logistic regression to examine the association between 30-day all-cause mortality and baseline characteristics in COVID-19 patients stepwise is shown in the Supplement 9. After adjusting for age and obesity, only stage-2 untreated and treated hypertension significantly increased odds of 30-day all-cause mortality, (OR = 2.64, 95%CI [1.18–5.90], p = 0.019) and (OR = 2.06, 95%CI (1.06–4.01), p = 0.034); respectively. If adjusting only for age and diabetes, only stage-2 untreated hypertension remained significant (OR = 2.39, 95%CI [1.05–5.46], p = 0.037). However, after adjustment for age, diabetes and obesity none of six JNC8 groups showed any significant difference in 30-day all-cause mortality (Supplement 9).

## Discussion

**Figure 1:**
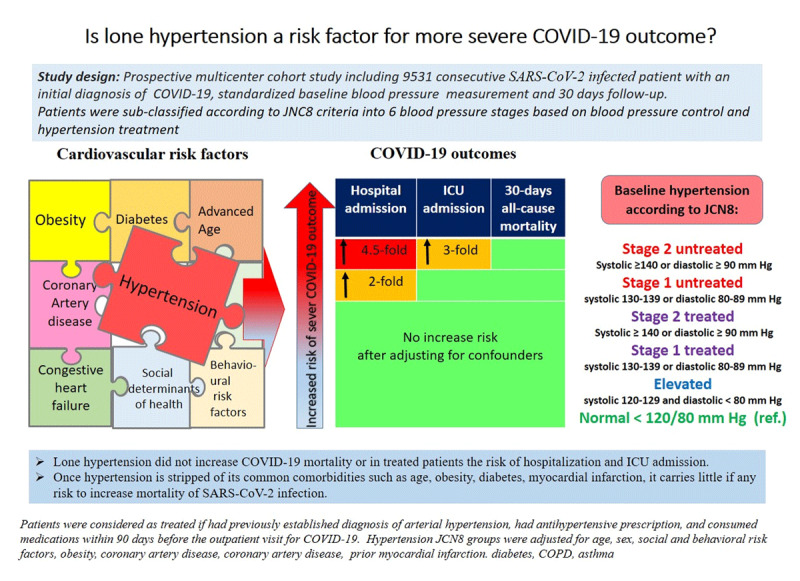
Lone hypertension and more severe course of COVID-19: This central illustration represents a summary of the study design, patients cohort, the study results and the conclusions.

In our study cohort, hypertension neither increased COVID-19 mortality nor the risk of hospitalization in patients with controlled BP. After adjusting for age, sex, coronary artery disease, history of myocardial infarction, type-2 diabetes, obesity, the presence of hypertension had no statistically significant effect of on 30-day all-cause mortality regardless of JNC8 BP stage (***Figure 1***). Thirty days after the onset of the first symptoms, 112 (1.18%) patients died. A total of 1,183 (12.4%) patients had to be hospitalized. Compared to the patients with normal BP, stage-2 untreated hypertension increased the odds for hospitalization 4.51-fold, stage 1 untreated hypertension 1.97-fold, and elevated BP 1.87-fold. The increased hospitalization rate in untreated BP groups remained significant after adjusting for confounders, such as age, sex, history of myocardial infarction, type-2 diabetes, and obesity. The reason for increased odds of hospitalization in patients with untreated hypertension is unclear. Conceivably the fact that hypertension has been associated with an increased risk of more severe outcome and fear of possible complications may have triggered hospitalization in some of these patients [15,16,17,18]. More than 1-in-8 patients who had their exams at the COVID-19 distribution centers were subsequently referred to COVID-19 hospitals. This relatively high rate might be explained by the ordinance of the Ministry of Health to dispatch people to the allocated COVID-19 hospitals if any increased risk for complications is expected, and by the fact that treatment was free of charge for these patients. Our results confirm increased odds with age, obesity, history of diabetes and CVD for more severe course, admission to the hospital and 30-day all-cause mortality.

In regard to antihypertensive treatment, a recently published systematic review and meta-analysis including 31 cohort studies provided outcome data for 87,951 patients with COVID-19, of whom 22,383 out of 83,963 (26.7%) were on ACEI/ARB therapy [18]. There was no differential effect for mortality/severe disease outcomes for patients who were on ACEI and ARB treatment, indicating that ACEI/ARB should not be discontinued. In our study, hypertension stage-2 untreated patients had a more severe course of the disease including longer duration of symptoms and higher hospitalization rates. A significant impact of obesity, diabetes, cardiovascular diseases on hospital admission and all-cause mortality emphasizes the importance of preventive measures to improve short-term and long-term outcomes for COVID-19 patients.

The strength of this study is that this is a prospective observational cohort study including a large number of consecutive Covid-19 confirmed cases with no loss of 30-day follow-up. We assessed the impact of major comorbidities (e.g. age, hypertension, obesity, diabetes, history of myocardial infarction) on the course of COVID-19, outcomes (such as hospitalization and ICU admission), as well as 30-day all-cause mortality. The first physician exam was within two days from onset of symptoms in 96% of participants. Established active follow-up daily monitoring by the healthcare providers allowed responding quickly and hospitalizing patients if complications have been expected.

There are several limitations related to this study. One limitation is the relatively young study population which does not allow to extrapolate the results to older populations. However, if sensitivity tests were conducted on 3,087 patients who were 50 years old and older, the statistical analysis did not show any significant difference for any stage of hypertension as well, thus the results are presented for the entire cohort. The decisions for hospitalizations were very subjective and influenced by the fact that hospital cost were fully covered by the government. The fact that at the time of the study, the presence of hypertension was considered a risk factor for a more serious disease course may have influenced diagnostic and therapeutic decisions. Finally, this is an observational cohort study and there may be confounders which we have not been taken into consideration.

## Conclusion

Hypertension by definition is a hemodynamic disorder. Lone hypertension did not increase COVID-19 mortality or in treated patients risk of ICU admission and hospitalization. Untreated hypertensive patients were at the increased risk of hospital admission. Once it is stripped of its common comorbidities such as age, obesity, diabetes, myocardial infarction, it carries little if any risk to increase mortality of SARS-CoV-2 infection.

## Data sharing

Data collected for the study, including deidentified individual participant data, a data dictionary defining each field in the set, and the statistical analysis plan will be made available to other researchers on request from the time of publication of this manuscript. Research proposals can besubmitted to the corresponding author (HS) at *hugo.saner@med.unibe.ch* and the first author (EVSh) at *evgeniya.v.shalaeva@gmail.com*.

## Additional File

The additional file for this article can be found as follows:

10.5334/gh.1099.s1 Sensitivity analyses to test whether lone hypertension is a risk factor for more severe COVID-19 outcomes. The differences in the outcomes between the presence vs. no hypertension per se is shown in Supplement 1–3, whether controlled or uncontrolled hypertension compared with no hypertension were associated with severe COVID-19 (Supplement 4–7). Additional regression analysis stratified by gender is presented in Supplement 8. Univariable and multivariable logistic regression to examine the association between 30-day mortality and baseline characteristics in COVID-19 patients is shown in Supplement 9.
