# Supplementary material for: Is Lone Hypertension a Risk Factor for More Severe COVID-19 Outcomes?
Source: Glob Heart. 2022 Mar 1;17(1):17. doi: 10.5334/gh.1099 (PMC8896243; doi:10.5334/gh.1099)
Supplement: Sensitivity analyses to test whether lone hypertension is a risk factor for more severe COVID-19 outcomes. — The differences in the outcomes between the presence vs. no hypertension per se is shown in Supplement 1–3, whether controlled or uncontrolled hypertension compared with no hypertension were associated with severe COVID-19 (Supplement 4–7). Additional regression analysis stratified by gender is presented in Supplement 8. Univariable and multivariable logistic regression to examine the association between 30-day mortality and baseline characteristics in COVID-19 patients is shown in Supplement 9. [file gh-17-1-1099-s1.pdf]

Supplement 1: Association between hospital admission and hypertension stratified presence vs no hypertension in COVID-19 patients adjusted for confounders, multivariable logistic regression (n=9531)

	Available data in the univariable analysis n=9531	Patients who were hospitalized n=1183	Univariable		Multivariable	
			Crude odds ratio (95% CI)	p value	Adjusted odds ratio (95% CI)	p value
**Arterial Hypertension**						
No	7089	474 (6.7)	1 (ref)		1 (ref)	..
Yes	2442	709 (29.0)	5.71 (5.02–6.49)	<0.001	1.38 (1.15–1.66)	0.001
**Age, years**	9531	1183	1.05 (1.05–1.06)	<0.001	1.026(1.02 –1.03)	<0.001
**Sex**						
Male	4689	545 (11.6)	1 (ref)		1 (ref)	..
Female	4842	638 (13.2)	1.15 (1.02–1.30)	0.018	1.11 (0.96–1.27)	0.160
**Myocardial infarction**						
No	8612	985 (11.4)	1 (ref)		1 (ref)	..
Yes	919	198 (21.5)	4.79 (3.98–5.78)	<0.001	1.18 (0.92–1.51)	0.182
**Obesity**						
No	7845	503 (6.4)	1 (ref)		1 (ref)	..
Yes	1686	680 (40.3)	9.86 (8.63–11.26)	<0.001	5.68 (4.91 –6.57)	<0.001
**Diabetes**						
No	8509	735 (8.6)	1 (ref)		1 (ref)	..
Yes	1022	448 (43.8)	8.26 (7.14–9.54)	<0.001	3.35 (2.78–4.04)	<0.001

Data are n (%) unless otherwise specified. CI = confidence interval.

Supplement 2: Association between ICU admission and hypertension stratified presence vs no hypertension in COVID-19 patients adjusted for confounders, multivariable logistic regression (n=9531)

	Available data in the univariable analysis n=9531	Patients admitted in the ICU n=132	Univariable		Multivariable	
			Crude odds ratio (95% CI)	p value	Adjusted odds ratio (95% CI)	p value
**Arterial Hypertension**						
No	7089	23 (0.32)	1 (ref)		1 (ref)	..
Yes	2442	109 (4.46)	14.35 (9.13–22.56)	<0.001	1.24 (0.72–2.10)	0.430
**Age, years**	9531		1.10 (1.09–1.12)	<0.001	1.07 (1.05 –1.09)	<0.001
**Sex**						
Male	4689	63 (1.3)	1 (ref)		1 (ref)	..
Female	4842	69 (1.4)	1.06 (0.75–1.49)	0.734	0.83 (0.57–1.22)	0.353
**Myocardial infarction**						
No	8612	88 (1.0)	1 (ref)		1 (ref)	..
Yes	919	44 (4.8)	9.09 (6.26–13.20)	<0.001	1.60 (1.01–2.52)	0.045
**Obesity**						
No	7845	27 (0.3)	1 (ref)		1 (ref)	..
Yes	1686	105 (6.2)	19.23 (12.56–29.45)	<0.001	6.02 (3.81–9.51)	<0.001
**Diabetes**						
No	8509	43 (0.5)	1 (ref)		1 (ref)	..
Yes	1022	89 (8.7)	18.78 (12.97–27.19)	<0.001	4.59 (3.01–6.99)	<0.001

Data are n (%) unless otherwise specified. CI = confidence interval. ICU = intensive care unit.

Supplement 3: Association between 30-days mortality and hypertension stratified presence vs no hypertension in COVID-19 patients adjusted for confounders, multivariable logistic regression (n=9531)

	**Available data in the univariable analysis n=9531**	**Patients who died n=112**	**Univariable**		**Multivariable**	
			Crude odds ratio (95% CI)	p value	Adjusted odds ratio (95% CI)	p value
**Arterial Hypertension**						
No	7089	16 (0.23)	1 (ref)		1 (ref)	..
Yes	2442	96 (3.9)	18.09 (10.63–30.77)	<0.001	1.01 (0.55–1.58)	0.982
**Age, years**	9531		1.13 (1.11–1.15)	<0.001	1.11 (1.08 –1.13)	<0.001
**Sex**						
Male	4689	48 (1.0)	1 (ref)		1 (ref)	..
Female	4842	64 (1.3)	1.29 (0.89–1.89)	0.178	0.97 (0.67–1.51)	0.922
**Myocardial infarction**						
No	8612	57 (0.7)	1 (ref)		1 (ref)	..
Yes	919	55 (5.9)	18.01 (12.29–26.38)	<0.001	2.67 (1.67–4.31)	<0.001
**Obesity**						
No	7845	18 (0.2)	1 (ref)		1 (ref)	..
Yes	1686	94 (5.6)	25.68 (15.46–42.63)	<0.001	7.65 (4.44 –13.17)	<0.001
**Diabetes**						
No	8509	31 (0.4)	1 (ref)		1 (ref)	..
Yes	1022	81 (7.9)	23.54 (15.48–35.81)	<0.001	4.40 (2.73–7.09)	<0.001

Data are n (%) unless otherwise specified. CI = confidence interval.

Supplement 4: Baseline characteristics of COVID-19 patients with the 30-days outcome (n=9531). Hypertension was subclassified as no hypertension, controlled, and uncontrolled.

	No Hypertension n=7089	Controlled Hypertension n=1344	Uncontrolled Hypertension n=1098	Total n=9531
**Age (years), mean ± SD**	36.1 ± 12.9	55.0 ± 10.8	64.9 ± 9.5	41.9 ± 9.7
**Age ≥ 65 years, n (%)**	232 (3.3)	214 (15.9)	513 (46.7)	959 (10.1)
**Sex, n (%)**				
Male	3574 (50.4)	578 (43.0)	528 (48.1)	4689 (49.2)
Female	3515 (49.6)	757 (56.3)	570 (51.9)	4842 (50.8)
**Course of COVID-19 disease**				
Mild	3862 (54.5)	504 (37.5)	354 (32.2)	4720 (49.5)
Moderately severe	2720 (38.4)	507 (37.7)	403 (36.7)	3630 (38.1)
Severe	484 (6.8)	296 (22.0)	269 (24.5)	1049 (11.0)
Critical (ICU admission)	23 (0.3)	37 (2.5)	72 (6.6)	132 (1.4)
**Comorbidities**				
**History of myocardial infarction**	146 (2.1)	140 (10.4)	245 (22.3)	534 (5.6)
**Obesity**	704 (9.9)	507 (37.7)	475 (43.3)	1686 (17.7)
**Diabetes**	265 (3.7)	332 (24.0)	425 (38.7)	1022 (10.7)
**COPD/Asthma**	513 (7.2)	221 (16.4)	185 (16.8)	919 (9.6)
**Antihypertensive medications:**				
ACEI	146 (2.1)	711 (52.9)	545 (49.6)	1387 (14.6)
ARB	72 (1.0)	338 (25.1)	430 (39.2)	840 (8.8)
CCB	42 (0.6)	162 (12.1)	170 (15.5)	374 (3.9)
BB	34 (0.5)	714 (53.1)	446 (40.6)	1192 (12.5)
Diuretics	38 (0.5)	188 (14.0)	104 (9.5)	330 (3.5)
**Outcomes:**				
**Duration of COVID-19, days#**	8.7 ± 6.0	21.9 ± 9.4	14.6± 8.2	10.4 ± 7.3
**Hospitalisation**	474 (6.3)	341 (62.5)	368 (25.9)	1183 (12.4)
Stay in hospital, days	9.5 ± 3.6	10.5 ± 4.3	11.8 ± 4.6	10.5 ± 4.2
Stay in ICU, days	3.0 ± 1.8	4.1 ± 2.8	5.1 ± 3.4	4.4 ± 3.1
**All-cause mortality 30-days**	16 (0.2)	23 (1.7)	73 (6.6)	112 (1.2)

Data are n (%), where n is the number of participants with non-missing data, or mean ± SD. Baseline characteristics were calculated for the participants at the first medical exam. #COVID-19 symptoms onset to recovery or symptoms onset to death. ACEI = Angiotensin-converting enzyme inhibitors. ARB = Angiotensin-receptor blockers. BB = Beta-blockers. CCB = Calcium channel blockers CI = confidence interval. ICU = intensive care unit.

Supplement 5: Association between <u>hospital admission</u> and <u>hypertension</u> stratified by no/controlled/uncontrolled hypertension in COVID-19 patients adjusted for confounders, multivariable logistic regression (n=9531)

	**Available data in the univariable analysis n=9531**	**Patients who were hospitalized n=1183**	**Univariable**		**Multivariable**	
			Crude odds ratio (95% CI)	p value	Adjusted odds ratio (95% CI)	p value
**Arterial Hypertension**						
No	7089	474 (6.7)	1 (ref)		1 (ref)	..
Controlled (<140/90mmHg)	1344	341 (25.7)	4.75 (4.06–5.53)	<0.001	1.42 (1.17–1.73)	<0.001
Uncontrolled (≥140/90mmHg)	1098	368 (33.5)	7.03 (6.02–8.22)	<0.001	1.31 (1.04–1.64)	0.023
**Age, years**	9531	1183	1.05 (1.05–1.06)	<0.001	1.02(1.02 –1.03)	<0.001
**Sex**						
Male	4689	545 (11.6)	1 (ref)		1 (ref)	..
Female	4842	638 (13.2)	1.15 (1.02–1.30)	0.018	1.1 (0.96–1.27)	0.107
**Myocardial infarction**						
No	8612	985 (11.4)	1 (ref)		1 (ref)	..
Yes	919	198 (21.5)	4.79 (3.98–5.78)	<0.001	1.19 (0.93–1.53)	0.058
**Obesity**						
No	7845	503 (6.4)	1 (ref)		1 (ref)	..
Yes	1686	680 (40.3)	9.86 (8.63–11.26)	<0.001	5.67 (4.91 –6.57)	<0.001
**Diabetes**						
No	8509	735 (8.6)	1 (ref)		1 (ref)	..
Yes	1022	448 (43.8)	8.26 (7.14–9.54)	<0.001	3.38 (2.79–4.07)	<0.001

Data are n (%), where n is the number of participants with non-missing data. CI = confidence interval.

Supplement 6: Association between admission to the intensive care unit and hypertension stratified by no/controlled/uncontrolled hypertension in COVID-19 patients adjusted for confounders, multivariable logistic regression (n=9531)

	Available data in the univariable analysis n=9531	Patients admitted in the ICU n=132	Univariable		Multivariable	
			Crude odds ratio (95% CI)	p value	Adjusted odds ratio (95% CI)	p value
**Arterial Hypertension**						
No	7089	23 (0.32)	1 (ref)		1 (ref)	..
Controlled (<140/90mmHg)	1344	37 (54.8)	8.69 (5.15–14.68)	0.573	1.20 (0.67–2.15)	0.309
Uncontrolled (≥140/90mmHg)	1098	72 (6.6)	21.56 (13.48–16.05)	0.408	1.27 (0.72–3.85)	0.165
**Age, years**	9531		1.10 (1.09–1.12)	<0.001	1.07 (1.05 –1.09)	<0.001
**Sex**						
Male	4689	63 (1.3)	1 (ref)		1 (ref)	..
Female	4842	69 (1.4)	1.06 (0.75–1.49)	0.734	0.83 (0.57–1.22)	0.441
**Myocardial infarction**						
No	8612	88 (1.0)	1 (ref)		1 (ref)	..
Yes	919	44 (4.8)	9.09 (6.26–13.20)	<0.001	1.59 (1.01–2.52)	0.016
**Obesity**						
No	7845	27 (0.3)	1 (ref)		1 (ref)	..
Yes	1686	105 (6.2)	19.23 (12.56–29.45)	<0.001	6.04 (3.82–9.54)	<0.001
**Diabetes**						
No	8509	43 (0.5)	1 (ref)		1 (ref)	..
Yes	1022	89 (8.7)	18.78 (12.97–27.19)	<0.001	4.57 (2.99–6.98)	<0.001

Data are n (%), where n is the number of participants with non-missing data. CI = confidence interval. ICU = intensive care unit.

Supplement 7: Association between 30-days mortality and hypertension stratified by no/controlled/uncontrolled hypertension in COVID-19 patients adjusted for confounders, multivariable logistic regression (n=9531)

	Available data in the univariable analysis n=9531	Patients who died n=112	Univariable		Multivariable	
			Crude odds ratio (95% CI)	p value	Adjusted odds ratio (95% CI)	p value
**Arterial Hypertension**						
No	7089	16 (0.22)	1 (ref)		1 (ref)	..
Controlled (<140/90mmHg)	1344	23 (1.7)	7.69 (4.05–14.61)	<0.001	0.77 (0.37–1.58)	0.476
Uncontrolled (≥140/90mmHg)	1098	73 (6.7)	31.48 (18.25–54.29)	<0.001	1.17 (0.62–2.21)	0.633
**Age, years**	9531		1.13 (1.11–1.15)	<0.001	1.10 (1.08 –1.13)	<0.001
**Sex**						
Male	4689	48 (1.0)	1 (ref)		1 (ref)	..
Female	4842	64 (1.3)	1.29 (0.89–1.89)	0.178	0.96 (0.62–1.47)	0.835
**Myocardial infarction**						
No	8612	57 (0.7)	1 (ref)		1 (ref)	..
Yes	919	55 (5.9)	18.01 (12.29–26.38)	<0.001	2.67 (1.67–4.31)	<0.001
**Obesity**						
No	7845	18 (0.2)	1 (ref)		1 (ref)	..
Yes	1686	94 (5.6)	25.68 (15.46–42.63)	<0.001	7.79 (4.52 –13.44)	<0.001
**Diabetes**						
No	8509	31 (0.4)	1 (ref)		1 (ref)	..
Yes	1022	81 (7.9)	23.54 (15.48–35.81)	<0.001	4.32 (2.67–6.96)	<0.001

Data are n (%), where n is the number of participants with non-missing data, or mean ± SD. CI = confidence interval.

Supplement 8: Multivariate logistic regression stratified by gender to determine the association between 30-days mortality and hypertension stratified by six JNC8 groups in COVID-19 patients adjusted for confounders (n=9531)

	**Male n=4689**	**Male who died n=48 (1.0%)**	Adjusted odds ratio (95% CI)	p value	**Female n=4842**	**Female who died n=64 (1.3%)**	Adjusted odds ratio (95% CI)	p value
**Arterial Hypertension**								
Normal	3347 (71.4)	7 (0.2)	1 (ref)		3350 (69.2)	6 (0.2)	1 (ref)	..
Elevated	305 (6.5)	5 (1.6)	2.61 (0.71–9.61)	0.150	263 (5.4)	3 (1.1)	2.29 (0.50–10.37)	0.284
Stage 1 untreated	209 (4.5)	0	0	0.995	248 (5.1)	0	0	0.995
Stage 1 treated	300 (6.4)	9 (3.0)	1.25 (0.41–3.84)	0.693	411 (8.5)	9 (2.2)	1.22 (0.37–3.97)	0.744
Stage 2 untreated	108 (2.3)	7 (6.5)	1.35 (0.42–4.39)	0.624	125 (2.6)	7 (5.6)	2.12 (0.65–7.38)	0.205
Stage 2 treated	420 (9.0)	20 (4.8)	0.81 (0.29–2.29)	0.697	445 (9.2)	39 (8.8)	2.27 (0.87–6.11)	0.099
**Age, years**	41.5±16.0	69.9±6.9	1.12 (1.08 –1.16)	<0.001	42.6±16.2	71.1±8.1	1.08 (1.05 –1.11)	<0.001
**Myocardial infarction**								
No	4429(94.5)	27 (0.6)	1 (ref)	..	4568 (94.3)	30 (0.7)	1 (ref)	..
Yes	260 (5.5)	21 (8.1)	2.35 (1.12–4.99)	0.026	274 (5.7)	34 (12.4)	3.26 (1.71–6.20)	<0.001
**Obesity**								
No	3888(82.9)	10 (0.3)	1 (ref)	..	3957 (81.7)	8 (0.2)	1 (ref)	..
Yes	801 (17.1)	38 (4.7)	7.33 (3.39–15.58)	<0.001	885 (18.3)	56 (6.3)	6.92 (3.06–15.58)	<0.001
**Diabetes**								
No	4196(89.5)	12 (0.3)	1 (ref)	..	4313 (89.1)	19 (0.4)	1 (ref)	..
Yes	493 (10.5)	36 (7.3)	4.70 (2.2–9.96)	<0.001	529 (10.9)	45 (8.5)	3.81 (2.0–7.24)	<0.001

Data are n (%), where n is the number of participants with non-missing data, or mean ± SD. CI = confidence interval.

Supplement 9: Association between 30-days mortality and hypertension subclassified into six JNC8 groups in COVID-19 patients adjusted for confounders, multivariable logistic regression (n=9531)

	Available data in the univariable analysis n=9531	Patients who died n=112	Odds ratio (95% CI)	p value	Chi-square for the model	-2 Log likelihood	Cox & Snell R Square	Nagelkerke R Square
**Arterial Hypertension unadjusted**					241.17	976.92	0.025	0.208
Normal	6697	13 (0.2)	1 (ref)					
Elevated	568	8 (1.4)	7.35 (3.03–17.79)	<0.001				
Stage 1 untreated	457	0	0	0.994				
Stage 1 treated	711	18 (2.5)	13.35 (6.52–27.37)	<0.001				
Stage 2 untreated	233	14 (6.0)	32.87 (15.27–70.76)	<0.001				
Stage 2 treated	865	59 (6.8)	37.63 (20.55–68.92)	<0.001				
**Arterial Hypertension adjusted for age, years**					388.96	829.13	0.040	0.333
Normal	6697	13 (0.2)	1 (ref)	..				
Elevated	568	8 (1.4)	5.83 (2.34–14.54)	<0.001				
Stage 1 untreated	457	0	0	0.993				
Stage 1 treated	711	18 (2.5)	2.75 (1.31–5.77)	0.007				
Stage 2 untreated	233	14 (6.0)	6.16 (2.82–13.42)	<0.001				
Stage 2 treated	865	59 (6.8)	3.39 (1.77–6.51)	<0.001				
**Age, years**	9531		1.12 (1.10 –1.14)	<0.001				
**Arterial Hypertension adjusted for history of prior Myocardial Infarction**					312.15	905.94	0.032	0.269
Normal	6697	13 (0.2)	1 (ref)	..				
Elevated	568	8 (1.4)	6.17 (2.53–15.03)	<0.001				
Stage 1 untreated	457	0	0	0.992				
Stage 1 treated	711	18 (2.5)	9.84 (4.74–20.42)	<0.001				

Stage 2 untreated	233	14 (6.0)	20.60 (9.33–45.49)	<0.001				
Stage 2 treated	865	59 (6.8)	19.77 (10.43–37.47)	<0.001				
**Myocardial infarction**								
No	8612	57 (0.7)	1 (ref)	..				
Yes	919	55 (5.9)	6.27 (4.15–9.48)	<0.001				
**Arterial Hypertension adjusted for Obesity**					357.87	860.21	0.037	0.307
Normal	6697	13 (0.2)	1 (ref)	..				
Elevated	568	8 (1.4)	2.7 (1.08–6.71)	0.032				
Stage 1 untreated	457	0	0	0.992				
Stage 1 treated	711	18 (2.5)	5.39 (2.56–11.37)	<0.001				
Stage 2 untreated	233	14 (6.0)	9.63 (4.31–21.52)	<0.001				
Stage 2 treated	865	59 (6.8)	14.40 (7.62–27.21)	<0.001				
**Obesity**								
No	7845	18 (0.2)	1 (ref)	..				
Yes	1686	94 (5.6)	11.89 (6.95–20.35)	<0.001				
**Arterial Hypertension adjusted for Diabetes**					344.54	873.55	0.036	0.296
Normal	6697	13 (0.2)	1 (ref)	..				
Elevated	568	8 (1.4)	5.12 (2.08–12.58)	<0.001				
Stage 1 untreated	457	0	0	0.992				
Stage 1 treated	711	18 (2.5)	6.55 (3.01–13.86)	<0.001				
Stage 2 untreated	233	14 (6.0)	8.94 (3.92–20.45)	<0.001				
Stage 2 treated	865	59 (6.8)	13.53 (7.02–26.08)	<0.001				
**Diabetes**								
No	8509	31 (0.4)	1 (ref)	..				
Yes	1022	81 (7.9)	9.20 (5.79–14.63)	<0.001				
**Arterial Hypertension adjusted for age and obesity**					488.86	729.24	0.050	0.417
Normal	6697	13 (0.2)	1 (ref)	..				

Elevated	568	8 (1.4)	2.81 (1.09–7.25)	0.032				
Stage 1 untreated	457	0	0	0.992				
Stage 1 treated	711	18 (2.5)	1.51 (0.71–3.12)	0.292				
Stage 2 untreated	233	14 (6.0)	**2.64 (1.18–5.90)**	**0.019**				
Stage 2 treated	865	59 (6.8)	**2.06 (1.06–4.01)**	**0.034**				
**Age, years**	9531		1.12 (1.09 –1.14)	<0.001				
**Obesity**								
No	7845	18 (0.2)	1 (ref)	..				
Yes	1686	94 (5.6)	9.98 (5.88–16.96)	<0.001				
**Arterial Hypertension adjusted for age and diabetes**					466.66	751.43	0.048	0.398
Normal	6697	13 (0.2)	1 (ref)	..				
Elevated	568	8 (1.4)	3.85 (1.49–10.03)	0.006				
Stage 1 untreated	457	0	0	0.992				
Stage 1 treated	711	18 (2.5)	1.87 (0.87–4.03)	0.109				
Stage 2 untreated	233	14 (6.0)	**2.39 (1.05–5.46)**	**0.037**				
Stage 2 treated	865	59 (6.8)	1.79 (0.9–3.56)	0.097				
**Age, years**	9531		1.11 (1.09 –1.14)	<0.001				
**Diabetes**								
No	7845	18 (0.2)	1 (ref)	..				
Yes	1686	94 (5.6)	6.89 (4.37–10.88)	<0.001				
**Arterial Hypertension adjusted for age, obesity and diabetes**					543.91	683.18	0.055	0.455
Normal	6697	13 (0.2)	1 (ref)	..				
Elevated	568	8 (1.4)	2.57 (0.96–6.88)	0.059				
Stage 1 untreated	457	0	0	0.992				
Stage 1 treated	711	18 (2.5)	1.25 (0.57–2.74)	0.584				
Stage 2 untreated	233	14 (6.0)	1.55 (0.67–3.57)	0.303				
Stage 2 treated	865	59 (6.8)	1.52 (0.77–3.02)	0.224				

**Age, years**	9531		1.11 (1.08 –1.14)	<0.001
**Obesity**				
No	7845	18 (0.2)	1 (ref)	..
Yes	1686	94 (5.6)	7.38 (4.03–12.66)	<0.001
**Diabetes**				
No	8509	31 (0.4)	1 (ref)	..
Yes	1022	81 (7.9)	4.59 (2.89–7.29)	<0.001
